# The broad spectrum mixed-lineage kinase 3 inhibitor URMC-099 prevents acute microgliosis and cognitive decline in a mouse model of perioperative neurocognitive disorders

**DOI:** 10.1186/s12974-019-1582-5

**Published:** 2019-10-28

**Authors:** Patrick Miller-Rhodes, Cuicui Kong, Gurpreet S. Baht, Priyanka Saminathan, Ramona M. Rodriguiz, William C. Wetsel, Harris A. Gelbard, Niccolò Terrando

**Affiliations:** 1 Center for Neurotherapeutics Discovery, University of Rochester Medical Center, Rochester, NY 14642 USA; 2 Department of Neuroscience, University of Rochester Medical Center, Rochester, NY 14642 USA; 3 Center for Translational Pain Medicine, Department of Anesthesiology, Duke University Medical Center, Durham, NC 27710 USA; 4 Department of Orthopedic Surgery and Duke Molecular Physiology Institute, Duke University Medical Center, Durham, NC 27710 USA; 5 Department of Microbiology and Immunology, University of Rochester Medical Center, Rochester, NY 14642 USA; 6 Department of Psychiatry and Behavioral Sciences, Mouse Behavioral and Neuroendocrine Analysis Core Facility, Duke University Medical Center, Durham, NC 27710 USA; 7 Departments of Neurobiology and Cell Biology, Duke University Medical Center, Durham, NC 27710 USA; 8 Department of Neurology, University of Rochester Medical Center, Rochester, NY 14642 USA; 9 Department of Pediatrics, University of Rochester Medical Center, Rochester, NY 14642 USA

**Keywords:** Microglia, Delirium, Postoperative neurocognitive disorders, Intravital microscopy, Mixed-lineage kinase 3

## Abstract

**Background:**

Patients with pre-existing neurodegenerative disease commonly experience fractures that require orthopedic surgery. Perioperative neurocognitive disorders (PND), including delirium and postoperative cognitive dysfunction, are serious complications that can result in increased 1-year mortality when superimposed on dementia. Importantly, there are no disease-modifying therapeutic options for PND. Our lab developed the “broad spectrum” mixed-lineage kinase 3 inhibitor URMC-099 to inhibit pathological innate immune responses that underlie neuroinflammation-associated cognitive dysfunction. Here, we test the hypothesis that URMC-099 can prevent surgery-induced neuroinflammation and cognitive impairment.

**Methods:**

Orthopedic surgery was performed by fracturing the tibia of the left hindlimb with intramedullary fixation under general anesthesia and analgesia. In a pilot experiment, 9-month-old mice were treated five times with URMC-099 (10 mg/kg, i.p.), spaced 12 h apart, with three doses prior to surgery and two doses following surgery. In this experiment, microgliosis was evaluated using unbiased stereology and blood-brain barrier (BBB) permeability was assessed using immunoglobulin G (IgG) immunostaining. In follow-up experiments, 3-month-old mice were treated only three times with URMC-099 (10 mg/kg, i.p.), spaced 12 h apart, prior to orthopedic surgery. Two-photon scanning laser microscopy and CLARITY with light-sheet microscopy were used to define surgery-induced changes in microglial dynamics and morphology, respectively. Surgery-induced memory impairment was assessed using the “What-Where-When” and Memory Load Object Discrimination tasks. The acute peripheral immune response to surgery was assessed by cytokine/chemokine profiling and flow cytometry. Finally, long-term fracture healing was assessed in fracture callouses using micro-computerized tomography (microCT) and histomorphometry analyses.

**Results:**

Orthopedic surgery induced BBB disruption and microglial activation, but had no effect on microglial process motility. Surgically treated mice exhibited impaired object place and identity discrimination in the “What-Where-When” and Memory Load Object Discrimination tasks. Both URMC-099 dosing paradigms prevented the neuroinflammatory sequelae that accompanied orthopedic surgery. URMC-099 prophylaxis had no effect on the mobilization of the peripheral innate immune response and fracture healing.

**Conclusions:**

These findings show that prophylactic URMC-099 treatment is sufficient to prevent surgery-induced microgliosis and cognitive impairment without affecting fracture healing. Together, these findings provide compelling evidence for the advancement of URMC-099 as a therapeutic option for PND.

## Introduction

Delirium and postoperative cognitive dysfunction, now collectively referred to as perioperative neurocognitive disorders (PND), have become the most common complications after routine surgical procedures, particularly in the elderly [1, 2]. Following surgery (e.g., common orthopedic procedures), up to 50% of patients experience cognitive disturbances that can lead to serious complications, including poorer prognosis and a higher 1-year mortality rate in subjects with pre-existing neurodegeneration [3]. PNDs have profound effects on our society and are estimated to incur tens of billions of dollars in health care costs annually [4]. Currently, there are no approved disease-modifying therapeutic options to treat PNDs and the pathogenesis of these complex phenomena is not fully understood.

To clarify the pathophysiology of PNDs, we have developed an orthopedic surgery mouse model in which the left hindlimb is subjected to an open tibial fracture with intramedullary fixation under general anesthesia and analgesia [5]. This model recapitulates features of clinical procedures such as a fracture repair or hip arthroplasty, which are often associated with neurological disorders in frail subjects. In this model, we and others have described a key role for surgery-induced neuroinflammation, including microgliosis [6–8], astrogliosis [9], inflammatory cell ingress [10], and hippocampal-dependent memory impairments [6, 9, 11, 12]. Imaging of [^11^C]PBR28 in patients receiving abdominal surgery revealed a role for microglial activation in the development of cognitive impairment in these individuals [13]. Notably, strategies aimed at depleting microglia before surgery abrogated neuroinflammation and subsequent cognitive decline in mice [10], but do not present realistic prophylactic treatment options for at-risk patients.

Using a variety of preclinical models of disease that feature neuroinflammation, including HIV-1-associated neurocognitive disorders (HAND) [14], Alzheimer’s disease (AD) [15, 16], and multiple sclerosis (MS) [17], we have demonstrated the therapeutic efficacy of the small-molecule, blood-brain barrier (BBB)-permeable, mixed-lineage kinase 3 (MLK3) inhibitor URMC-099 [15, 16]. In addition to exhibiting favorable pharmacokinetic and toxicity profiles in rodents, URMC-099 possesses “broad-spectrum” activity due to its ability to inhibit multiple kinases, and consequently downstream signaling pathways, that mediate pathological interactions between innate immune cells and end-organ target cells (such as neurons) [14, 17–19]. Although URMC-099 and other immunomodulatory approaches can reduce untoward microglial responses in a variety of chronic neuroinflammatory settings, they can pose translational concerns in the context of acute perioperative recovery, such as impairing normal healing and tissue regeneration after surgical trauma.

Here, we test the hypothesis that prophylactic URMC-099 treatment can attenuate neuroinflammation and PND-like deficits using an established model of orthopedic surgery-induced PND. Using a combination of two-photon laser scanning microscopy (2P-LSM) and translationally relevant behavioral paradigms, we show that prophylactic URMC-099 treatment reduces pathological changes in microglial morphology while preventing memory decline. Finally, we observed no effects of URMC-099 treatment on the acute peripheral immune response and long-term bone healing following orthopedic surgery. Thus, our findings support the development of URMC-099 for prophylactic treatment of PND.

## Materials and methods

### Animals

C57BL/6 (Jax stock no. 000664) and CX3CR1-GFP (Jax stock no. 005582) mice were obtained from Jackson Laboratories (Bar Harbor, ME). Heterozygous male and female CX3CR1-GFP mice (3 months of age) were used for the longitudinal two-photon laser-scanning microscope (2PLSM) experiment. Male and female C57BL/6 mice (3 months of age) were used for the cytokine/chemokine profiling and flow cytometry experiment. Male C57BL/6 mice, at 3 and 9 months of age, were used for the remainder of the experiments as indicated. All animal procedures were carried out under protocols approved by the Duke University Medical Center and University of Rochester Medical Center, Institutional Animal Care and Use Committee, under the National Research Council Guide for the Care and Use of Laboratory Animals, 8th edition. Both Duke University and University of Rochester Medical Center are AAALAC-accredited institutions.

### Orthopedic surgery model

Orthopedic surgery was performed as described previously [5]. Briefly, the left hindlimb was subjected to an open tibial fracture with intramedullary fixation under general anesthesia (isoflurane: 4% induction, 2% maintenance) and analgesia (buprenorphine: 0.1 mg/kg, s.c.). Sham-treated mice were administered anesthesia and analgesia as above but without surgical intervention. Analgesia was either administered in a sustained release format or re-administered twice daily for each experiment as needed.

### URMC-099 administration

**Fig. 1:**
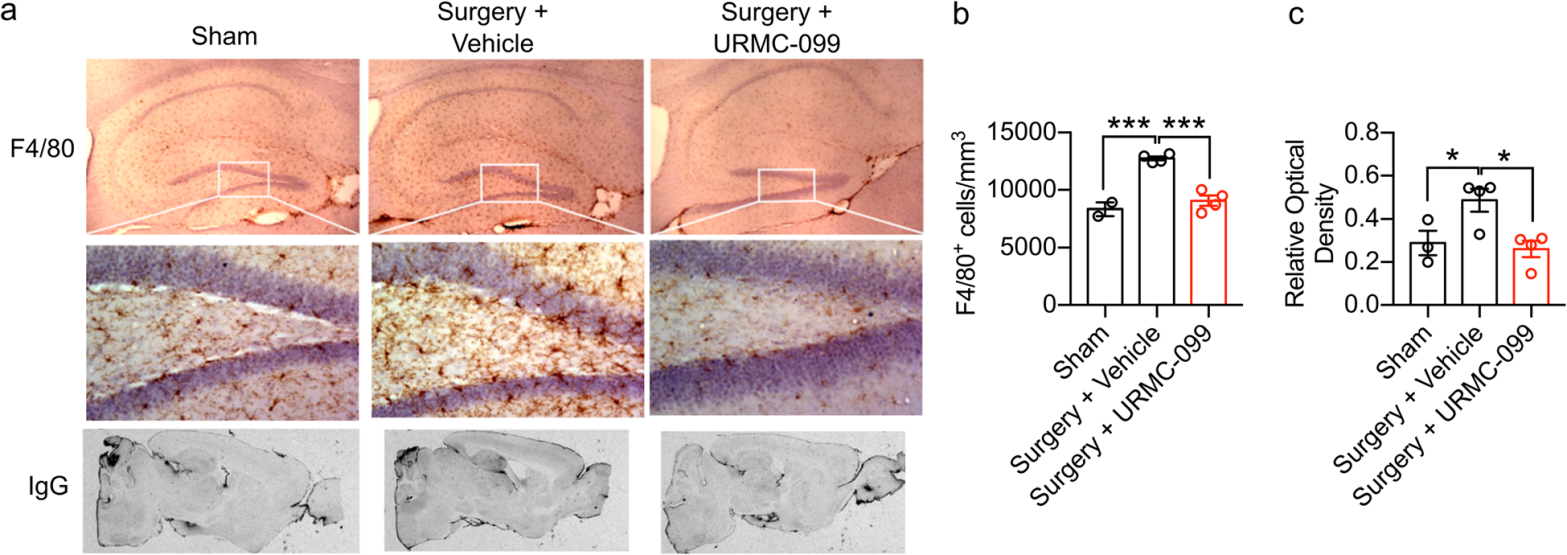
URMC-099 treatment reverses microgliosis and BBB leak following orthopedic surgery. Nine-month-old male mice underwent open tibial fracture and were terminated 24 h after surgery. URMC-099 was given i.p. at 10 mg/kg, three doses before surgery and two doses after surgery based on Marker et al. [14]. **a** Representative images of F4/80 microglial/macrophage staining (top panels) and IgG staining (bottom panels) show that URMC-099 significantly attenuated microgliosis in the hippocampus. **b** Differences in microglia cell numbers were quantified by unbiased stereology. **c** IgG relative optical density was quantified to measure differences in BBB opening. *N* = 2–4; results presented as mean ± SEM; *P < 0.05, ***P < 0.001, versus surgery + vehicle group; one-way ANOVA with Dunnett’s multiple comparison test

URMC-099 (M.W. 421) was synthesized as originally described in Goodfellow et al. [18]. URMC-099 drug solutions were prepared by dissolving 20 mg of URMC-099 in 0.5 mL sterile dimethyl sulfoxide (DMSO; D8779, Sigma-Aldrich, St. Louis, MO). We then added 4 mL polyethylene glycol 400 (PEG400; 91893-250-F, Sigma-Aldrich) and 5.5 mL sterile saline (National Drug Code NDC0409-4888-10). The final concentration was 2 mg/mL URMC-099 in a 10 mL volume. The vehicle was the same solution minus URMC-099. For the experiments described in Fig. 1, mice were administered (i.p.) three injections at 12 h intervals prior to orthopedic surgery and two injections post-surgery at identical intervals. For all other experiments, mice were treated only with three injections (spaced 12 h apart) prior to surgery. Drug solutions were coded such that experimenters were blinded to the experimental conditions for the duration of the experiments.

### Immunohistochemistry and stereological analysis

Immunohistochemistry (IHC) and stereological analysis of F4/80+ cells in the dorsal hippocampus were performed as described previously [20]. Briefly, 9-month-old mice were perfused transcardially with PBS and immersion-fixed in 4% formaldehyde overnight. Following transfer to a sucrose gradient, 40 μm sections were cut using a cryostat. IHC was performed on free-floating sections using a monoclonal rat anti-mouse F4/80 primary antibody (1:10,000; Serotec, Raleigh, NC, USA) and a biotinylated goat anti-rat immunoglobulin G (IgG) secondary antibody (1:3000; Vector Laboratories, Inc., Burlingame, CA, USA), after which sections were treated with avidin-biotin-peroxidase complex (ABC kit; Vector Laboratories, Inc., Burlington, CA, USA). Staining was visualized using diaminobenzidine (DAB; Vector Laboratories) and counterstained with hematoxylin (Fisher Scientific, Fair Lawn, NJ, USA). Stereological counting was performed using a Nikon 218912 light microscope interfaced with the StereoInvestigator software package (MicroBrightField, Williston, VT, USA). IgG leakage was assessed in adjacent sections (5 per mouse) as above except we used a biotinylated anti-mouse IgG antibody (1:3000).

### Piezoelectric thinned-skull cortical window surgery

The thinned-skull cortical window (TSCW) procedure was performed using a piezoelectric surgical technique [21] to enable 2PLSM of microglia in CX3CR1-GFP mice. Piezoelectric surgery was performed using a piezosurgery handpiece (Mectron) fitted with an OT11 osteotomy insert (Mectron) under constant, manual irrigation (1 mL/min) with ice-cold artificial cerebrospinal fluid to minimize the heating of the bone during the thinning process. A cover glass was glued over the thinned area of the skull, and a custom head-plate was placed with dental cement to facilitate repeated intravital imaging. Isoflurane was used for anesthesia (4% induction, 2% maintenance) during the TCSW procedure. The TCSW surgery was limited to 30 min or less to minimize the mouse’s exposure to variables that might confound this study’s results (e.g., duration under anesthesia).

### Two-photon intravital microscopy

A custom two-photon laser-scanning microscope was used for intravital imaging (Ti:Sapphire, Mai-Tai, Spectraphysics; modified Fluoview confocal scan head, × 20 lens, 0.95 numerical aperture, Olympus). Two-photon excitation was achieved using 100-fsec laser pulses (80 MHz) tuned to 840 nm with a power of ∼ 50 mW measured at the sample after the objective lens. For all experiments, a 565-nm dichroic mirror was used in conjunction with 580/180 (GFP) and 605/55 (rhodamine B) filters. Rhodamine B dextran (2% w/v, 75 μL total) was injected retro-orbitally after ketamine (100 mg/kg)/xylazine (10 mg/kg) anesthesia and 5 min prior to intravital imaging. Mice were maintained at 37 °C during intravital imaging and recovery. Importantly, intravital imaging sessions were limited to 40 min per mouse to avoid re-exposure to ketamine/xylazine anesthesia. Intravital imaging was performed using × 4 digital zoom and at a 512 × 512-pixel resolution. Stacks containing 41 slices (1 μm step size) were imaged every 90 s for 15 min to obtain the XYZT stacks used for analysis.

### CLARITY tissue processing and light-sheet microscopy

Brains were harvested following transcardial perfusion with 30 to 50 mL of PBS (#10010-023; Gibco) and 20 to 30 mL 4% paraformaldehyde (PFA) (#158127; Sigma-Aldrich) in PBS (pH 7.4) under isoflurane anesthesia. After a post-fixation step (24 h in 4% PFA at 4 °C), brains were sectioned by vibratome into 1-mm-thick coronal sections. Sections were incubated in 1× PBS overnight at 4 °C prior to polymerization and tissue clearing. Polymerization and tissue clearing were accomplished using the X-CLARITY™ reagents and equipment (Logos Biosystems). Sections were first saturated in 1 mL hydrogel solution (X-CLARITY™ Hydrogel Solution Kit, #C1310X, Logos Biosystems) at 4 °C for 24 h and were subsequently incubated in the polymerization system for 3 h at 37 °C. Polymerized sections were then actively cleared in electrophoretic tissue clearing (ETC) solution (#C13001, Logos Biosystems) using the X-CLARITY™ Tissue Clearing System with the following settings: 0.9 A, 37 °C, for 3 h. Cleared sections were washed overnight three times with 0.2% Triton X-100 (T8787, Sigma) in PBS on a shaker at room temperature. Immunohistochemistry was performed by incubating cleared tissue sections with rabbit anti-Iba1 (1:300; cat no. 019-19741; Wako) in 10% normal donkey serum + 0.2% Triton X-100 in PBS (blocking buffer) at 4 °C for 3 days. Following three wash steps (0.1% Triton X-100 in PBS, overnight at 4 °C), sections were incubated with Alexa Fluor 594-conjugated donkey anti-rabbit secondary antibody (1:500) and DAPI diluted in blocking buffer for 3 days at 4 °C. Sections were subsequently stored in PBS until mounting.

For imaging, sections were mounted in 1% agarose (#BP160-100, Fisher Scientific) and hung in 1 mL syringes (#300013, BD Biosciences) without tips followed by incubation with mounting solution (#C13101, Logos Biosystems) at 4 °C overnight. Samples were placed at room temperature for 1 h before imaging. Imaging was performed using a light-sheet fluorescence microscope (LSFM, Z1, Zeiss, Germany) at the Duke University Light Microscopy Core Facility. Imaging was performed by suspending samples with mounting solution in a CLARITY-optimized sample chamber and orienting the samples such that they were parallel to the detection lens. XYZ stacks were acquired at 1920 × 1920 pixel resolution and 16-bit depth using × 5 or × 20 objectives. The refractive index of the detection objective was set as 1.46. A 405/488/561/640 filter set was used for laser excitations with laser intensities set between 0.5 and 2%. An exposure time of 29.97 ms for each frame was used, and a step size of 0.659 μm was used. Three-dimensional surface reconstructions of microglia were achieved using Imaris as described below.

### Imaris image analysis

Individual microglia were cropped from XYZT stacks, registered, and drift-corrected using ImageJ. Individual microglia XYZT stacks were imported into Imaris (Bitplane) for analysis. For motility analysis, the “Spots” function was used to assign spots to individual microglial processes. Spots were assigned automatically using an estimated diameter of 2 μm, which were subsequently filtered by “Quality” and again manually to remove Spots that did not correspond to the individual microglia under analysis. The “Connected Components” algorithm was then used to generate the movement tracks that were quantified. Movement tracks with a duration of less than 3 min were excluded from analysis. For morphological analysis, the “Surface” function was used. Surface detail was set to 0.691 μm. Surfaces were then generated by manual thresholding and were manually filtered to exclude surfaces that did not correspond to the individual microglia under analysis. Sphericity—defined as the ratio of the area of a sphere (with the same volume as the given particle) to the surface area of the particle—was used to quantify microglial morphology. Similar measures have been used previously to quantify changes in microglia morphology, albeit in two dimensions [22]. Three-dimensional surface reconstructions of microglia were also performed on XYZ stacks derived from CLARITY tissue sections using the “Surface” function as described above with the following modifications: individual microglia were not cropped (instead, whole XYZ stacks were reconstructed) and surface detail was set to 2 μm to facilitate image processing.

### Cytokine/chemokine profiling and flow cytometry

Whole blood was collected from the retro-orbital sinus at 6 h post-surgery. EDTA (0.5 M) was used as an anticoagulant. Blood samples were centrifuged at 2000 rpm at 4 °C to isolate plasma for cytokine/chemokine profiling. Proteome Profiler cytokine arrays (R&D Systems) were used to measure cytokines and chemokines in 100 μL plasma/mouse according to the manufacturer’s instructions. RBC lysis was performed on the remaining blood pellet using ACK lysing buffer (Gibco), after which live cells were stained with Ghost Violet 510 viability dye (Tonbo Biosciences) for 30 min at 4 °C. Fc receptors were blocked with unconjugated anti-CD16/32 (Tonbo Bioscience) for 15 min on ice prior to staining with the following antibodies: APC-ef780-labeled CD45 (Clone 30-F11, eBioscience), PE-efluro 610-labeled CD11b (Clone: M1/70, Biolegend), PECy5-labeled CD11c (Clone: N418, Biolegend), AF647-conjugated Ly6C (Clone: HK1.4, Biolegend), and BV605 Ly6G (Clone: 1A8, BD Bioscience) for 30 min on ice in the dark. Subsequently, samples were washed and fixed with 2% paraformaldehyde for 15 min at room temperature. The BD FACSCanto II Flow Cytometer was used to measure fluorescence. The gating strategy for this experiment is presented in Additional file 1: Figure S1.

### Behavioral testing

Mice used for behavioral experiments were all housed in a temperature- and humidity-controlled environment, in Optimice ventilated cages (Animal Care Systems, Centennial, CO) with free access to water and mouse chow (5R58 Mouse Pico Diet, LabDiet, St. Louis, MO) under a 14-h light to 10-h dark-light cycle with the onset of light at 0700 h. For all behavioral tests, mice were transferred to the test room immediately following surgery and were housed in this room for the duration of the study. All test rooms were maintained under the same conditions as the colony housing room. Two memory tasks were administered to assess different aspects of memory performance 24 h following orthopedic surgery. The first task, a “What-Where-When” Object Recognition Test [23], was used to evaluate different aspects of episodic memory. This task involves three phases designated “set A”, “set B”, and “challenge.” Mice were habituated to a 60 × 40 × 24-cm arena for 5 min. For set A, mice were returned to the arena to explore four identical objects placed in configuration A (Fig. 4a) for 5 min. Animals were removed and placed into their home cages for an inter-trial interval (ITI) of 50–55 min. For set B, mice returned to the arena and were exposed to configuration B objects (Fig. 4a) for 5 min. The Challenge trial was administered after a 50–55-min ITI and included two set A and two B objects arranged in a sequence reflecting the “What”, “Where”, and “When” aspects of episodic memory (Fig. 4a). All behavioral trials were recorded by a video camera suspended above the test arena that was interfaced to a computer running Media Player II (Noldus Information Technology, Asheville, NC). Videos were scored in a blinded fashion using behavioral recognition and nose-point tracking with Ethovision 11 (Noldus Information Technology, Asheville, NC). These variables included duration of contacts within each object zone (contact = nose within 1 cm of the physical object and with the body axis oriented toward the object). “What”, “Where”, and “When” memory indices were computed from exploration times in the Challenge trial. The “What” aspect of episodic memory was defined as the mean exploration time for both A objects minus the mean exploration time for both B objects over the mean exploration time of all objects. The “What” aspect takes advantage of the animal’s preference for exploring the less recently experienced (A objects) over those more recently experienced (B objects). The “Where” aspect was defined as the mean exploration time for displaced object A minus the mean exploration time for stationary object A over the mean exploration time of both A objects. The “When” aspect was defined as the mean exploration time for “oldest” stationary object A minus the mean exploration time for the two most recent B objects over the mean exploration time for all three of these objects. The “When” index takes advantage of mice exploring the less recent “A” object over the more recent “B” objects while these objects all remain in their original locations.

Memory load was evaluated using the Memory Load Object Discrimination test [24]. This task was conducted in a 42 × 42 × 20-cm arena, and it consisted of seven trials (ranging from 2 to 5 min, depending on the number of objects present); each trial was separated by an ITI of 1–2 min. On each subsequent trial, a unique novel object was placed into the arena together with the previously presented objects (i.e., the “familiar” objects). Thus, the arena contained one object in trial 1, two objects in trial 2, etc. The objects used in the test are depicted in Fig. 5b. Trial lengths were increased as the number of objects was increased: 2 min (trials 1–3), 3 min (trials 4–5), and 5 min (trials 6–7). For each trial, the newly added object was designated as the “target” object. Time spent exploring the target object versus all other objects was recorded as described above for the “What-Where-When” task. Memory preference scores for each trial were computed as ratios of the mean exploration time for the target object minus the mean exploration time for all other objects over the mean exploration time for all objects.

### Analysis of fracture healing

Fracture calluses were assessed as previously described [25]. Samples were dissected 21 days after injury (from mice used for the behavioral experiments) and fixed in 10% Zn-formalin at room temperature for 5 days. Micro-computed tomography (microCT) analysis was conducted using a Scanco vivaCT 80 (Scanco Medical, Brüttisellen, Switzerland) at a scan resolution of 8 μm. Calluses were scanned 1 mm proximal and 1 mm distal from the fracture site and assessed for total volume (TV) and bone volume (BV) in mm^3^, and bone mineral density (BMD) in mg HA/mm^3^. Fixed fracture calluses were decalcified using 12% EDTA pH 7.4, cleared of EDTA, and embedded into paraffin. Sections were cut at a thickness of 5 μm and stained using safranin-O/fast-green to visualize bone and cartilage. A minimum of five sections was used to conduct computer-assisted histomorphometry analysis, and results were presented as an amount relative to the total area of the fracture callus.

### Statistics

All data were analyzed by GraphPad PRISM (GraphPad Software, San Diego, CA) and represented as mean ± SEM. Bone healing data were analyzed by unpaired, two-tailed *t* tests. The remainder of the data was analyzed by one-way or repeated-measures ANOVA with Dunnett’s or Tukey’s multiple comparison tests as indicated in the text for each experiment. Statistical significance is defined as *P* < 0.05 throughout.

## Results

### URMC-099 treatment reverses microgliosis and BBB leak following orthopedic surgery

Microgliosis and BBB opening accompany cognitive deficits in mice after orthopedic surgery [5–7, 9, 11]. We evaluated whether URMC-099 treatment could reverse these pathological changes in a pilot experiment using 9-month-old male mice. Mice were treated with three intraperitoneal (i.p.) injections of URMC-099 (10 mg/kg) prior to and two injections over the next day following surgery, with all injections spaced 12 h apart. We selected this administration paradigm for our initial experiment because it effectively inhibited phosphorylation of whole brain c-Jun N-terminal kinase (JNK), a kinase downstream of MLK3, in an in vivo model of HAND [18]. At 24 h post-surgery, hippocampal microgliosis was observed in vehicle-treated mice receiving orthopedic surgery relative to sham-treated controls using stereological analyses of F4/80+ cells (*P* = 0.0003; Fig. 1a, b). In contrast, URMC-099-treated mice exhibited a significant reduction (*P* = 0.0003) in the density of F4/80+ cells in the hippocampus relative to the sham-treated controls (Fig. 1b), indicating that our treatment paradigm effectively inhibited surgery-induced hippocampal microgliosis. BBB integrity was examined also using IgG immunostaining. We observed significantly less IgG leakage in URMC-099-treated mice receiving surgery and sham-treated controls when compared to vehicle-treated mice receiving surgery (*P* values ≤ 0.044; Fig. 1c). Hence, URMC-099 was efficacious in preventing both microgliosis and leakage at the BBB.

### URMC-099 prophylaxis prevents microglial morphological changes following orthopedic surgery

**Fig. 2:**
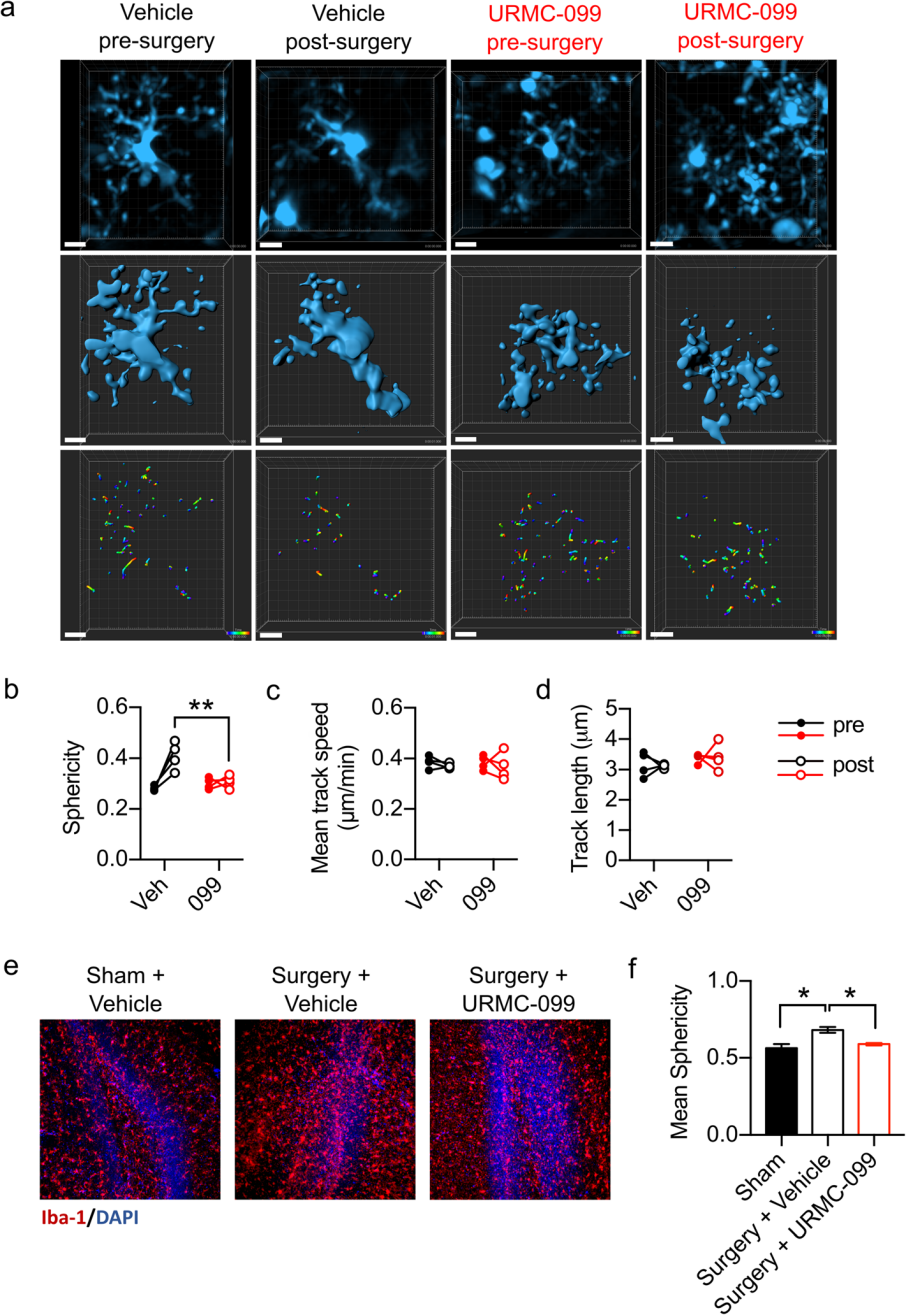
URMC-099 prophylaxis *prevents* microglial morphological changes following orthopedic surgery. Three-month-old mice received three doses i.p. of URMC-099 (10 mg/kg) prior to undergoing sham or orthopedic surgery. **a** Representative cropped and drift-corrected XYZ stacks obtained by 2PLSM (top panels), Imaris 3D surface reconstructions (middle panels), and process movement tracks (bottom panels) (scale bar = 10 μm). **b** Orthopedic surgery increased microglial sphericity in vehicle-treated, but not URMC-099-treated, mice. No differences were observed for mean track speed (**c**) nor mean track length (**d**). **e** Representative light-sheet micrographs obtained from optically cleared hippocampal sections. **f** Mean microglial sphericity was increased by orthopedic surgery, and URMC099 treatment prevented this. *N* = 4 (**b**–**d**), *N* = 2–3 (**f**); results presented as mean ± SEM; *P < 0.05, **P < 0.01, versus surgery + vehicle group; repeated-measures ANOVA (**b**–**d**) or one-way ANOVA (**f**) with Dunnett’s multiple comparison test

To follow-up on our initial findings, we next tested whether URMC-099 prophylaxis—pre-treatment with three injections of URMC-099 (10 mg/kg, i.p.), spaced 12 h apart, with the last dose occurring an hour before surgery—is sufficient to *prevent* microglial activation following orthopedic surgery. Because other researchers have observed changes in microglial morphology and process motility following a systemic inflammatory stimulus [26, 27], we used two approaches to define surgery-induced changes in microglial physiology and to test URMC-099’s ability to prevent these changes. One approach included longitudinal two-photon laser scanning microscopy (2PLSM) to define the morphology and dynamics of microglia in the superficial layers of the somatosensory cortex. A second involved light-sheet microscopy of CLARITY-processed hippocampal sections to define possible global changes in microglial morphology within this brain area. Longitudinal 2PLSM was performed by imaging vehicle or URMC-099-treated mice immediately prior to and 24 h post-orthopedic surgery and was achieved using a modified, Piezosurgical thinned-skull cortical window (TSCW) technique [21]. Images from vehicle-treated mice exhibited a reduction in the process complexity of microglia post-surgery compared to their pre-surgery images, as quantified by cell “sphericity”, or the normalized ratio of a microglia’s volume to its surface area (Fig. 2a, b). This measure is analogous to a previously validated ramification index used to assess microglia morphology changes [22]. Notably, URMC-099 prophylaxis abrogated this post-surgical effect, as microglia from URMC-099-treated mice remained mostly unchanged between their pre- and post-surgery images (*P* = 0.0016; Fig. 2a, b). This result was replicated on a more global scale using CLARITY (Fig. 2e, f; Additional file 2: Video S1, Additional file 3: Video S2, and Additional file 4: Video S3). We evaluated also microglial process motility over the course of 15 min of time-lapse imaging. However, we detected no significant differences in microglial process speed or track length between pre- and post-surgery images irrespective of treatment (Fig. 2c, d). Finally, we did not observe any BBB permeability following retro-orbital injection of rhodamine B dextran (MW 70,000 kDa) throughout our 2PLSM experiments (Additional file 5: Figure S2).

### URMC-099 prophylaxis does not inhibit the peripheral innate immune response

**Fig. 3:**
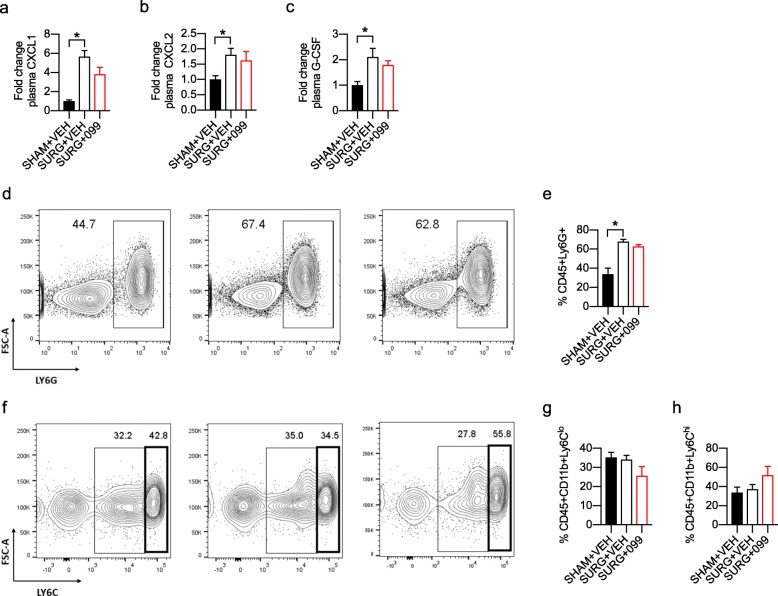
URMC-099 prophylaxis does not inhibit the peripheral innate immune response. Three-month-old mice received three doses i.p. of URMC-099 (10 mg/kg) prior to undergoing sham or orthopedic surgery. Whole blood was isolated 6 h post-surgery and used for cytokine/chemokine profiling and flow cytometry. **a**–**c** URMC-099 prophylaxis had no effect on surgery-induced plasma G-CSF, CXCL1, and CXCL2 levels at 6 h post-surgery. **d**–**e** URMC-099 prophylaxis had no effect on the surgery-induced increase in neutrophils at 6 h post-surgery. **f**–**h** URMC-099 prophylaxis and surgery did not significantly alter inflammatory (**g**) and patrolling monocyte (**h**) populations at 6 h post-surgery. *N* = 4; results presented as mean ± SEM; *P < 0.05, versus surgery + vehicle group; one-way ANOVA with Dunnett’s multiple comparison test

Although URMC-099 penetrates the BBB, URMC-099 prophylaxis could prevent microglial activation by inhibiting the peripheral innate immune response to surgery. To assess this, we treated male and female mice prophylactically with URMC-099 or vehicle and isolated blood via the retro-orbital sinus 6 h post-surgery. From these blood samples, we separated plasma and white blood cells for cytokine/chemokine profiling and flow cytometric analysis, respectively. We used Proteome Profiler mouse cytokine arrays to quantify changes in plasma chemokines and cytokines with surgery and URMC-099 treatment (Additional file 6: Figure S3). We observed strong induction of three analytes in our vehicle-treated surgical group relative to sham controls: C-X-C motif chemokine ligand 2 (CXCL1; ~ 5-fold, *P* = 0.0004; Fig. 3a), C-X-C motif chemokine ligand 2 (CXCL2; ~ 1.75-fold, *P* = 0.0489; Fig. 3b), and granulocyte-colony stimulating factor (G-CSF; ~ 2-fold, *P* = 0.0144; Fig. 3c). Together, these analytes mediate neutrophil expansion and trafficking during tissue inflammation [28, 29]. However, we observed no effect of URMC-099 treatment on the plasma levels of these analytes following surgery, although CXCL1 was marginally lower in URMC-099-treated surgical mice relative to their vehicle-treated counterparts (*P* = 0.0794; Fig. 3a). Surprisingly, this assay did not detect changes in pro-inflammatory cytokines in our samples. As a complementary measure, we further evaluated the peripheral immune response by flow cytometry of leukocytes derived from the same blood samples. We used several surface markers to identify various innate immune cell populations, including neutrophils (CD45+Ly6G+), inflammatory monocytes (CD45+Ly6C^hi^), patrolling monocytes (CD45+Ly6C^lo^), and CD8 T cells (CD45+CD8+). In agreement with our Proteome Profiler results, we observed a robust increase in the neutrophil population with surgery (*P* = 0.0005; Fig. 3d, e) that was not mitigated by URMC-099 prophylaxis. In contrast, we did not observe any significant differences in patrolling (Fig. 3f, g) or inflammatory (Fig. 3f, h) monocyte populations between any of our groups. Overall, we observed no effect of URMC-099 on the acute peripheral immune response 6 h following orthopedic surgery.

### URMC-099 prophylaxis abrogates orthopedic surgery-induced memory deficits

**Fig. 4:**
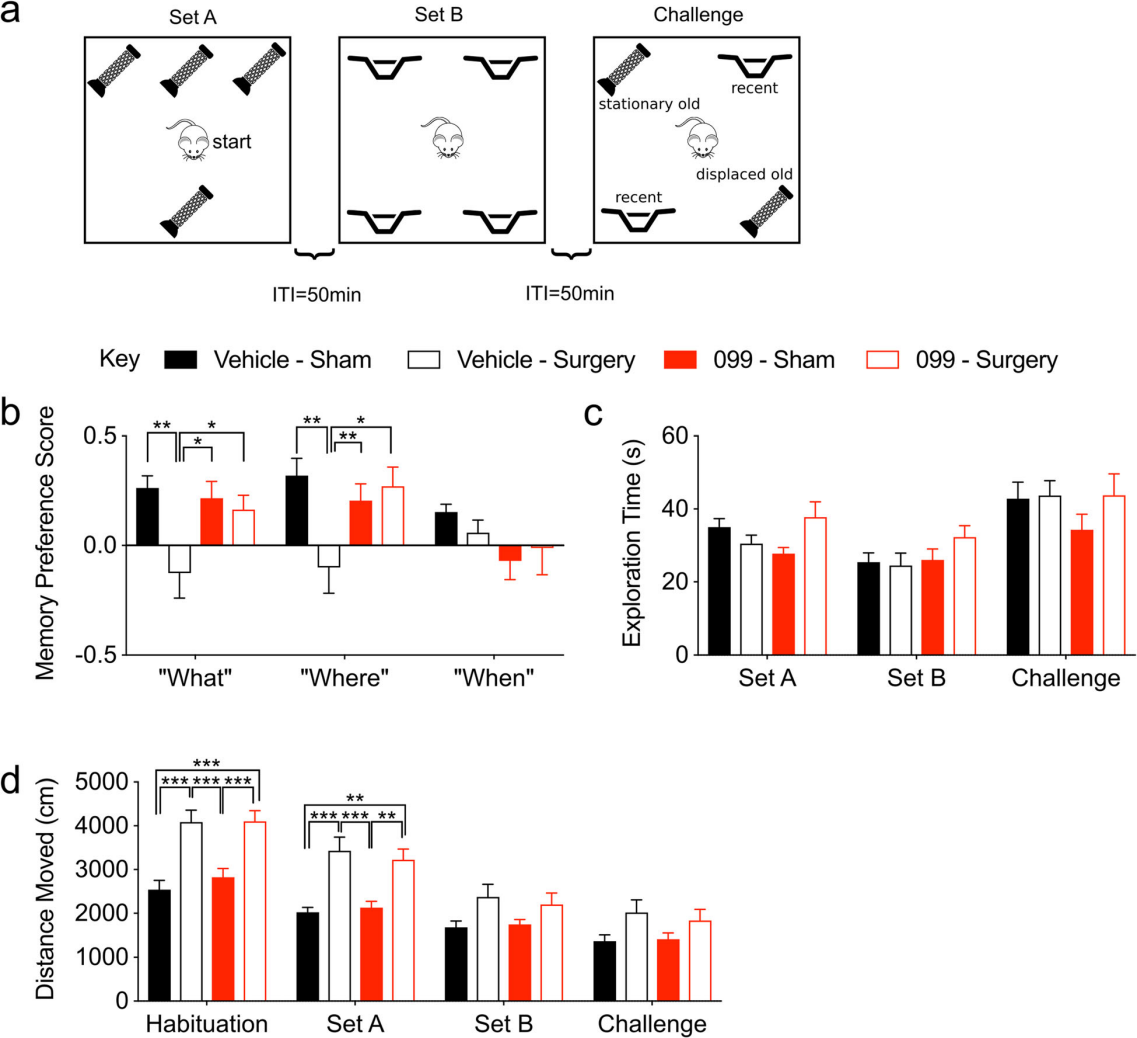
URMC-099 prophylaxis abrogates orthopedic surgery-induced deficits in object place and identity discrimination. Three-month-old mice received three doses i.p. of URMC-099 (10 mg/kg) prior to undergoing sham or orthopedic surgery. **a** An illustration of the “What-Where-When” Object Discrimination test. **b** URMC-099 prevented the surgery-induced impairments in the “What” and “Where” phases of the test, whereas “When” was unaffected. **c** Neither surgery nor drug treatment affected the total exploration times throughout the task. **d** Orthopedic surgery increased the total distance traveled during “set A” relative to sham-treated controls for both the vehicle- and URMC-099-treated mice. *N* = 10; results presented as mean ± SEM; *P < 0.05, **P < 0.01, ***P < 0.001, comparisons as indicated; repeated-measures ANOVA with Dunnett’s multiple comparison test (memory preference scores) or Tukey’s multiple comparison test (distance moved)

We evaluated cognitive dysfunction in our orthopedic surgery model using two translationally relevant behavioral tasks that probe hippocampal-dependent memory: the “What-Where-When” task [23] and the Memory Load Object Discrimination task [24]. We tested for cognitive impairment beginning 24 h post-surgery. As such, our behavioral paradigm models acute, delirium-like cognitive deficits, similar to those observed in a prospective cohort study of elderly orthopedic surgical patients [30]. The “What-Where-When” task tests various components of episodic memory in mice by requiring mice to remember which objects they explored (“What”), their object location (“Where”), and the temporal phase when they were explored (“When”) (Fig. 4a). Orthopedic surgery induced memory impairments in vehicle-treated mice in the “What” (*P* = 0.005) and “Where” (*P* = 0.007) aspects of the task (Fig. 4b). Additionally, we found that URMC-099 prevented the surgery-induced memory impairment in the “Where” (*P* = 0.0183) and “What” (*P* = 0.0432) aspects of the task (Fig. 4b). No differences in performance were detected among our experimental conditions in the “When” aspect of the task. Although it is unclear why this memory process was not affected, it could be due to the low levels of performance across all groups in this task.

To ensure that our behavioral data reflect cognitive deficits, we compared the object contact times and motor performance during each phase of the “What-Where-When” task. The object contact times were similar across groups at each of the three phases of the task, indicating that the deficiencies of the vehicle sham group on the “What” and “Where” phases of the task cannot be attributed to an inability to interact with objects (Fig. 4c). Surprisingly, there was a main effect of treatment on distance moved during this task [*F* (3, 144) = 24.60, *P* < 0.0001] (Fig. 4d). Post hoc comparisons revealed that both the vehicle- and URMC-099-treated groups that received orthopedic surgery traveled over greater distances than their sham counterparts (*P* values ≤ 0.0037) during habituation and the set A phase of the task (Fig. 4d). While the increased locomotion observed in surgical mice could reflect behavioral agitation caused by postoperative pain, this is unlikely for a number of reasons. First and foremost, we administered analgesia to mitigate postoperative pain with known efficacy up to 72 h. Second, while we observed significant differences in behavioral performance between vehicle- and URMC-099-treated surgical mice, we observed no differences between these two groups in the distance traveled during each phase. If URMC-099’s effect on behavioral performance reflected an analgesic effect, it would likely manifest as *reduced* distance moved specifically in the URMC-099-treated surgical group relative to the vehicle-treated surgical group during habituation and set A. Third, the effect of surgery on locomotion was only present during habituation and set A, which could instead reflect differences in anxiety behaviors between sham and surgical mice. Neither secondary measure was significantly different between any of our groups during the “Challenge” phase of this task. To more completely rule out a relationship between the distance moved during habituation/set A and behavioral performance in the surgical mice, we correlated these variables for each component of the task (i.e., “What”, “Where”, and “When”). None of these comparisons revealed significant correlations (Additional file 7: Figure S4). Overall, these data indicate (1) that orthopedic surgery does not significantly affect the ability of mice to perform the necessary locomotion to interact with objects in this task and (2) that their behavioral performance reflects impairments in object discrimination rather than agitation resulting from postoperative pain.

**Fig. 5:**
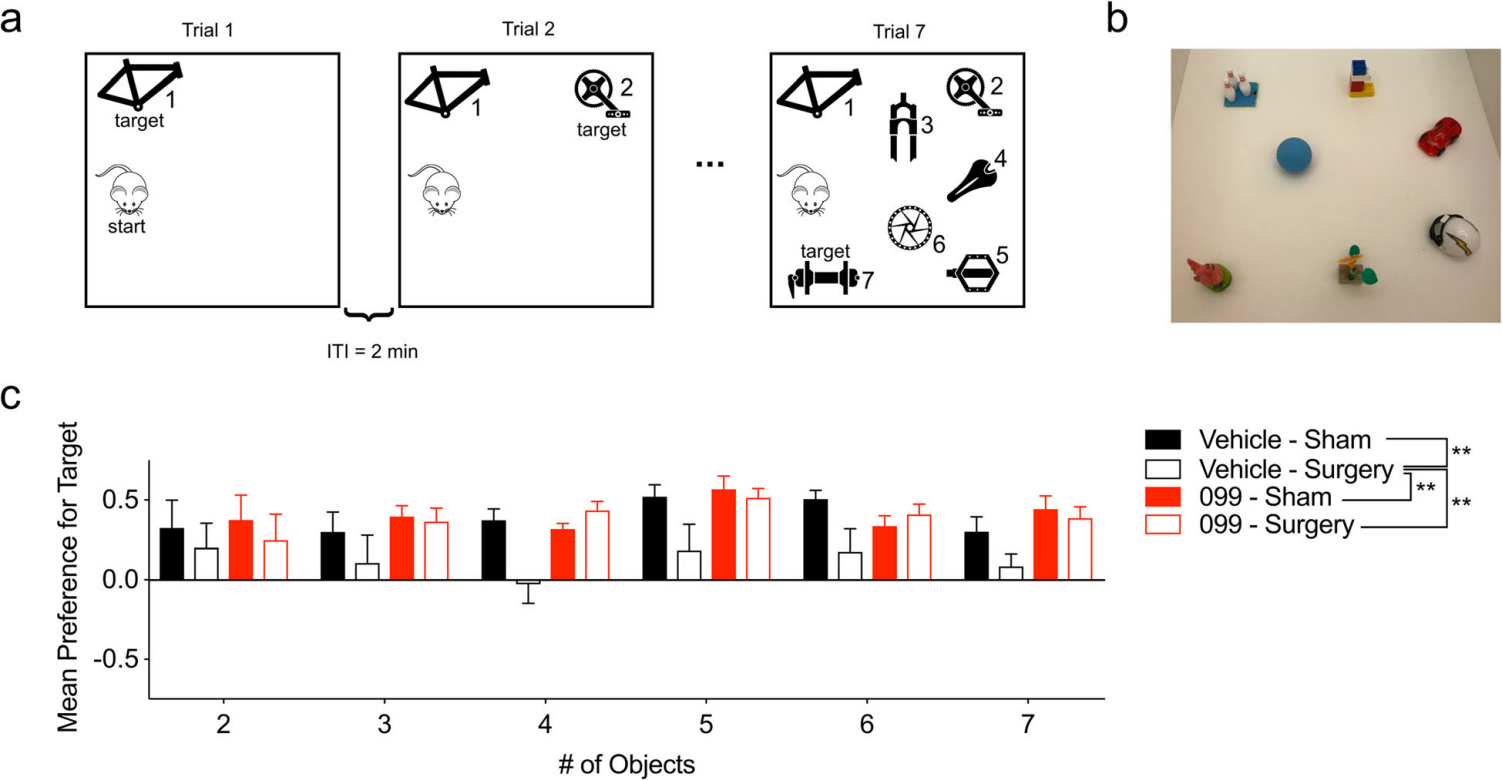
URMC-099 prophylaxis abrogates orthopedic surgery-induced deficits in the Memory Load task. Three-month-old mice received three doses i.p. of URMC-099 (10 mg/kg) prior to undergoing sham or orthopedic surgery. **a** An illustration of the Memory Load Object Discrimination test. **b** Illustration depicting the objects used for the task. **c** URMC-099 prevented the surgery-induced impairments in the Memory Load test overall. *N* = 10; results presented as mean ± SEM; *P < 0.05, **P < 0.01, versus surgery + vehicle group; repeated-measures ANOVA with Dunnett’s multiple comparison test

We further evaluated memory processes using a novel Memory Load Object Discrimination task [24]. In this modified novel object discrimination task, mice are presented with a single object during the first trial, and then, additional objects are subsequently added to the testing arena on each subsequent trial until the mouse encounters a total of seven objects (Fig. 5a, b). After trial 1, each subsequent trial tests the mouse’s ability to discriminate a novel object from the familiar object(s). Hence, this test evaluates how many items the mouse can hold in its memory before it can no longer recognize the novel object. Using RMANOVA, we identified a significant main effect of treatment on memory preference score in this task [*F* (3, 36) = 6.719, *P* = 0.001]. Overall, vehicle-treated mice that received surgery performed significantly worse overall relative to the vehicle-treated (*P* = 0.003) and URMC-099-treated (*P* = 0.0015) sham controls (Fig. 5c). Importantly, URMC-099 treatment prevented the object discrimination impairment in mice receiving surgery (*P* = 0.0025) (Fig. 5c). Given the lack of a main effect on trial (*P* = 0.1813), the impairments we observed in vehicle-treated surgical mice likely reflect a general impairment in object discrimination rather than deficient memory load. Taken together, the results from our behavioral studies indicate that the hippocampal-dependent impairments in “What” and “Where” memories and memory load in the surgical vehicle controls can be prevented by pretreatment with URMC-099.

### URMC-099 pre-treatment does not alter bone fracture healing

To evaluate whether prophylactic URMC-099 adversely affected bone healing in the surgically treated mice, we harvested fracture calluses 21 days post-fracture and used microCT and histomorphophometry analyses to quantify bone healing. The microCT assessment found no differences in bone mineralization between the surgical-vehicle and surgical URMC-099 groups (Additional file 8: Figure S5a–c), and histomorphometry analysis also confirmed an absence of group differences in bone deposition (Additional file 8: Figure S5d-e).

## Discussion

The present study demonstrates URMC-099’s therapeutic efficacy in a mouse model of orthopedic surgery-induced PND and adds to the growing literature documenting URMC-099’s neuroprotective effects [14, 15, 17, 18]. URMC-099 was discovered initially in search of potent MLK3 inhibitors with favorable CNS penetration properties. Previously, we characterized URMC-099’s kinase selectivity using a kinome-wide scanMAX assay, which revealed that URMC-099 potently inhibits tens of kinases with > 99% inhibition at 1 μM [18]. A few of these targets—notably, AXL, LRRK2, and FLT3—have been associated with microgliosis in various neuroinflammatory conditions [19, 31–33]. In particular, we have shown previously that LRRK2 inhibition exhibits neuroprotective effects similar to URMC-099 in a mouse model of HIV-1-associated neurocognitive disorders [19]. Thus, the results of the present study cannot be ascribed solely to inhibition of MLK3. Instead, we contend that URMC-099’s broad-spectrum activity is necessary to resolve the cognitive dysfunction we observed in our PND model.

Various factors, including age, endotoxin exposure, and diet-induced obesity, can exacerbate the neuroinflammatory sequelae associated with peripheral surgery [8, 10, 34, 35]. These effects can be explained in part by immunological training, or priming [36]. Immunological training is an epigenetic phenomenon whereby an immune cell’s “memory” of a previous event influences its subsequent reaction to another event. Indeed, a single injection of LPS (8 mg/kg) 5 days prior to partial hepatolobectomy exacerbated the effect of surgery on microglial activation [35]. Conversely, we have shown previously that a single injection of LPS (1 mg/kg) 24 h post-surgery similarly exacerbates the microglial response to peripheral surgery [8]. Additionally, microglia in aged mice are transcriptomically and functionally distinct from those in young mice [37–40], which is likely to affect their response to peripheral surgery. In future studies, beyond the scope of the present work, it will be interesting to compare the transcriptional responses of microglia to peripheral surgery in young and old mice.

The systemic milieu contributes to microglial activation and the cognitive decline that occurs with normal aging [41–45], which could also explain the exacerbated effects of surgery in aged mice. Systemic factors found in young blood have been shown to promote vascular remodeling, neurogenesis, and cognitive performance in aged mice [45]. Human umbilical cord plasma possesses similar rejuvenating properties [43]. Conversely, aged blood contains factors that negatively regulate cognitive function [41]. The deleterious effects of the aged systemic milieu—including both microglial activation and cognitive deficits—were recently shown to be mediated by brain endothelial cells in the absence of BBB breakdown [44]. Others have shown that stimulation of brain endothelial cells with IL-1 leads to IL-1 production in microglia [46]. Together, these studies suggest that brain endothelial cells can transmit signals from the systemic circulation that directly impact microglia and cognitive function *even without opening of the BBB*. Although we observed BBB disruption in older mice following orthopedic surgery, it is also possible that peripheral surgery induces microglial activation and impairs cognition via actions on brain endothelial cells, particularly in younger mice. In support of this, we did not observe BBB permeability after retro-orbital rhodamine B-dextran injection during our 2PLSM experiments. It is presently unknown whether URMC-099 exerts effects on brain endothelial cells, although we did observe a reduction in BBB leak in older, URMC-099-treated surgical mice.

This study is the first to use 2PLSM to examine how orthopedic surgery alters microglial morphology and dynamics. Our results indicate that orthopedic surgery induces morphological changes in microglia without overtly affecting microglial process motility. Although we are the first to examine microglial process motility in the context of surgical trauma, studies that utilize intraperotineal (i.p.) lipopolysaccharide (LPS) injection to induce microglial activation have neuroimmune similarities in that microglial activation is elicited rapidly via a peripheral inflammatory stimulus. Several groups have examined microglial process motility following i.p. LPS injection [26, 27, 47]. Most recently, Paris and colleagues [26] observed a minor, but statistically significant, decrease (0.25-fold) in process velocity 24 h post-LPS injection (1 mg/kg). Importantly, the use of isoflurane during imaging in this study may have had untoward effects on microglial motility via inhibition of microglial THIK-1 channels [22]. In stark contrast, Gyoneva and colleagues [27] saw increased process velocity 48 h post-LPS injection (2 mg/kg). A third study using a lesser dose of LPS (0.5 mg/kg) observed no changes in process motility 48 h post-LPS injection [47]. Thus, several factors—including the severity of the inflammatory stimulus, the timing of observation, and the method used to measure process velocity—could explain the discrepancy between these studies and ours. It is also possible that surgical trauma and peripheral LPS injection elicit distinct effects on microglial physiology by, for example, impinging on different innate immune signaling pathways. In support of this notion, genetic knockout of *Tlr4* does not prevent cognitive decline in mice receiving orthopedic surgery [11].

A primary goal of the present study was to evaluate the ability of URMC-099 to prevent surgery-induced memory impairment using behavioral tasks with translational relevance to PND. The two tasks we used—the “What-Where-When” task and the Memory Load Object Discrimination test—probe aspects of episodic memory and rely on intact functioning of the hippocampus and prefrontal cortex [23, 24]. Importantly, these tasks test aspects of cognitive impairment that have been implicated in a prospective cohort study of elderly orthopedic surgical patients [30]. Our observation that orthopedic surgery impaired performance in both the “What” and “Where” aspects of the “What-Where-When” task as well as the Memory Load Object Discrimination task implicates hippocampal dysfunction as a driver of memory impairment, as mice with hippocampal lesions perform poorly in both of these tasks [23, 24]. These results are consistent with our data showing microglial activation and BBB dysfunction in the hippocampus following orthopedic surgery. Although URMC-099 was able to prevent these impairments, the neurological substrate (e.g., synapse loss or synaptic dysfunction) of such impairments is still unknown. Although we did not examine long-term cognitive impairment in our surgically treated mice, the memory impairments we observed resemble those associated with delirium in human patients, which include deficits in working memory.

A major concern regarding the use of anti-inflammatory drugs for PND is whether they will adversely affect non-cognitive endpoints, such as fracture healing. This has been observed in other preclinical PND studies, where administration of a selective α7 subtype nicotinic acetylcholine receptor (α7 nAChR) agonist during the perioperative period abrogated orthopedic surgery-induced cognitive dysfunction, but also interfered with endochondral bone formation in an in vitro assay of bone remodeling [8]. Further, fracture healing depends upon the early production of pro-inflammatory cytokines [48] and the recruitment of inflammatory cells that mediate distinct phases of fracture repair [49, 50]. In fact, treatment with low-dose recombinant TNF-α has been shown to improve bone healing by enhancing the mobilization of innate immunity to the fracture site [50]. Despite URMC-099’s ability to directly interfere with pro-inflammatory cytokine expression [14, 18], we observed no effect of URMC-099 on bone healing nor the mobilization of innate immunity in the periphery in this study. In agreement with these findings, we previously found no effect of URMC-099 prophylaxis on the infiltration of peripheral cells into the CNS in a mouse model of HIV-1-associated neurocognitive disorders [14]. Alternatively, anti-inflammatory drugs that *do* interfere with bone healing may exert their detrimental effects through multiple mechanisms that extend beyond the initial mobilization of innate immunity, including direct effects on chondrogenic differentiation [8, 51]. Nonetheless, although MLK3 activation is known to regulate bone mineralization [52], we contend that our prophylactic, time-limited treatment paradigm helped to preclude any detrimental effect of our drug on long-term bone repair.

## Conclusion

Overall, the present study further defines the neurocognitive sequelae that accompany orthopedic surgery and emphasizes the therapeutic efficacy of URMC-099’s broad-spectrum activity in a mouse model of PND. Our recently published results with URMC-099 in a mouse model of experimental autoimmune encephalitis suggest that this compound may be therapeutically advantageous to a more highly selective MLK3 inhibitor [17]. Together, our current and previous results suggest that inhibiting multiple kinase signaling pathways in parallel or downstream from MLK3 may be necessary to effect disease-modifying outcomes for neuroinflammatory conditions, including PND.

## Supplementary information

**Additional file 1:** **Figure S1.** Flow cytometry gating strategy. Debris and doublets were removed based on their forward and side scatter properties. Live single cells (negative for Ghost Violet 510) were used for further analysis. Leukocytes (CD45+ cells) were analyzed for their Ly6G expression. All Ly6G+ cells were identified as Neutrophils while the Ly6G- population was further gated based on the CD11b expression to separate the myeloid (CD11b+) from the lymphoid (CD11b-) population. Inflammatory monocytes were then identified as CD11b+, Ly6C Hi cells while CD11b+, Ly6C Low cells were classified as patrolling monocytes.

**Additional file 2:** **Video S1.** Representative video depicting Iba1 immunoreactivity in a 1 mm thick hippocampal section cleared using CLARITY from a sham-treated mouse. All mice were 3-month-old for this experiment. Iba1 is denoted in red; DAPI in blue (related to Figure 2).

**Additional file 3:** **Video S2.** Representative video depicting Iba1 immunoreactivity in a 1 mm thick hippocampal section cleared using CLARITY from a vehicle-treated surgery mouse. All mice were 3-month-old for this experiment. Iba1 is denoted in red; DAPI in blue (related to Figure 2).

**Additional file 4:** **Video S3.** Representative video depicting Iba1 immunoreactivity in a 1 mm thick hippocampal section cleared using CLARITY from a URMC-099-treated surgical mouse (Video S3). All mice were 3-month-old for this experiment. Iba1 is denoted in red; DAPI in blue (related to Figure 2).

**Additional file 5:** **Figure S2.** Representative Z-projections of intact, rhodamine B-labeled vasculature during 2PLSM acquisition post-surgery for vehicle- (left) and URMC-099-treated (right), 3-month-old mice.

**Additional file 6:** **Figure S3.** Images of Proteome Profiler cytokine arrays related to Figure 3. A key depicting the position of each analyte on each array is provided (top).

**Additional file 7: Figure S4.** In surgical mice, distance moved during training is not correlated with behavioral performance in the “What-Where-When” object discrimination task (related to Figure 4). Pearson correlations; number of XY pairs per comparison = 20 (10 URMC-099 + Surgery, 10 Vehicle + Surgery). R^2^- and *P*-values (two-tailed) are shown for each correlation.

**Additional file 8: Figure S5.** URMC-099 pre-treatment does not alter bone fracture healing. 3-month-old male mice were treated with either with URMC-099 or vehicle and fractures were induced surgically. Fracture calluses were investigated 21 days post-fracture to assess bone healing. **(a-c)** MicroCT was used to assess bone volume per total callus volume and bone mineral density. **(d-e)** Histomorphometry was used to determine the percent bone tissue within the fracture callus. *N*=6-7; results presented as mean ± SEM. Data analyzed by unpaired two-tailed t-test.

## Data Availability

The datasets used and/or analyzed during the current study are available from the corresponding author on reasonable request.

## Abbreviations

2PLSM: Two-photon laser-scanning microscope
BBB: Blood-brain barrier
i.p.: Intraperitoneal
LPS: Lipopolysaccharide
MLK3: Mixed-lineage kinase 3
PND: Perioperative neurocognitive disorders
TSWC: Thinned-skull cortical window
