# Supplementary figures and images for: The broad spectrum mixed-lineage kinase 3 inhibitor URMC-099 prevents acute microgliosis and cognitive decline in a mouse model of perioperative neurocognitive disorders
Source: J Neuroinflammation. 2019 Oct 28;16:193. doi: 10.1186/s12974-019-1582-5 (PMC6816182; doi:10.1186/s12974-019-1582-5)

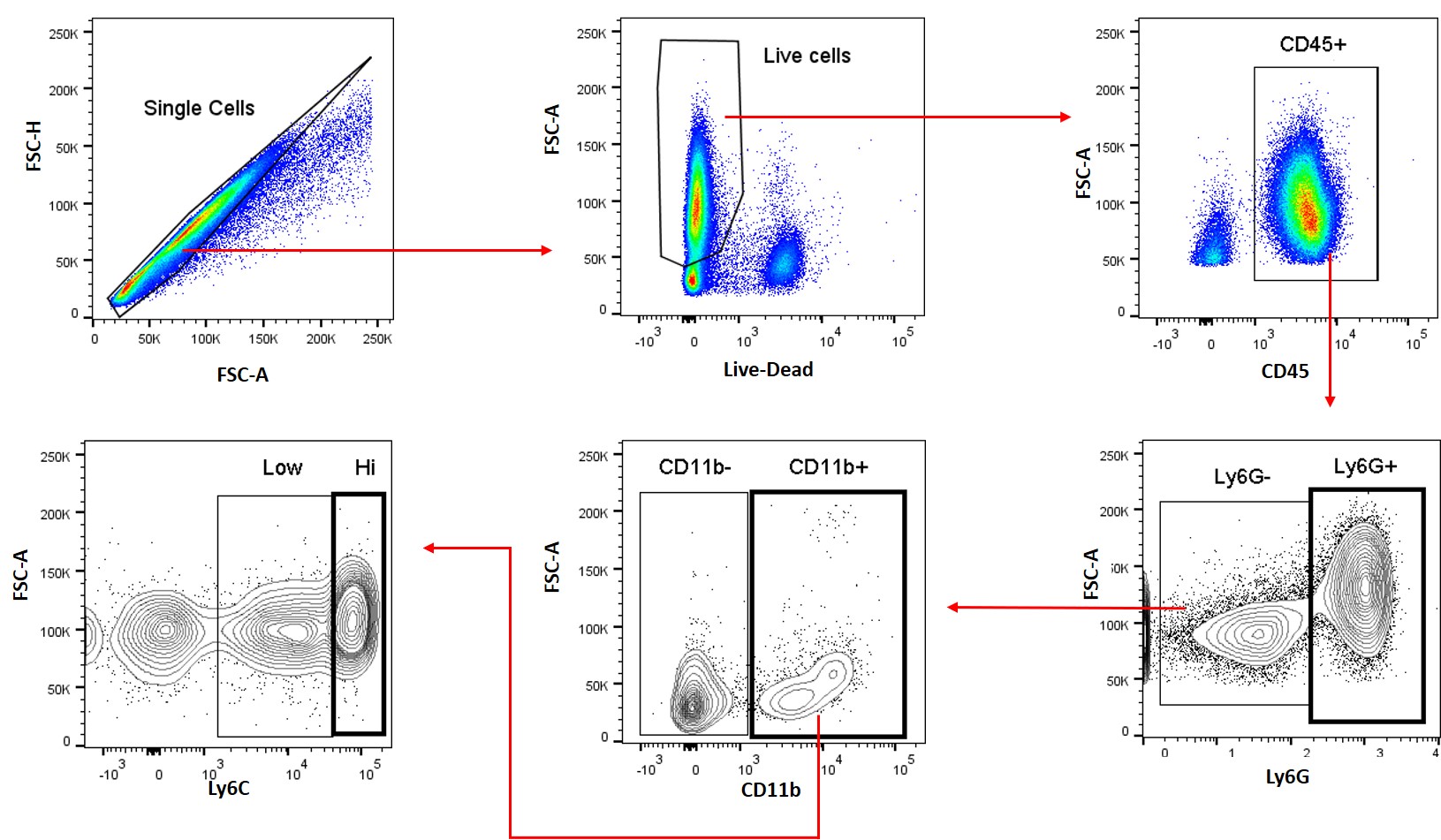

Supplement: Supplementary file 1 — Additional file 1: Figure S1. Flow cytometry gating strategy. Debris and doublets were removed based on their forward and side scatter properties. Live single cells (negative for Ghost Violet 510) were used for further analysis. Leukocytes (CD45+ cells) were analyzed for their Ly6G expression. All Ly6G+ cells were identified as Neutrophils while the Ly6G- population was further gated based on the CD11b expression to separate the myeloid (CD11b+) from the lymphoid (CD11b-) population. Inflammatory monocytes were then identified as CD11b+, Ly6C Hi cells while CD11b+, Ly6C Low cells were classified as patrolling monocytes. [file 12974_2019_1582_MOESM1_ESM.jpg]

Vehicle, post-surgery

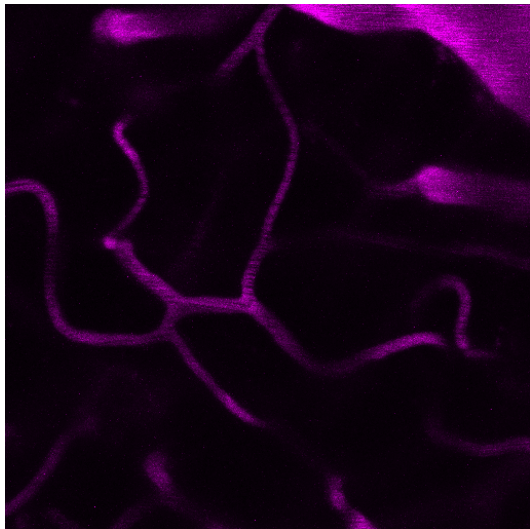

URMC-099, post-surgery

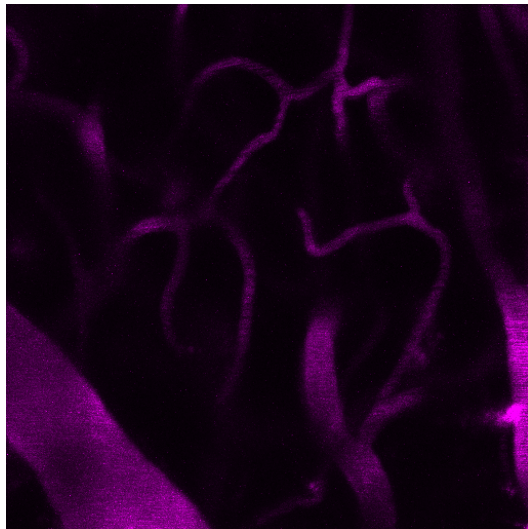

Supplement: Supplementary file 5 — Additional file 5: Figure S2. Representative Z-projections of intact, rhodamine B-labeled vasculature during 2PLSM acquisition post-surgery for vehicle- (left) and URMC-099-treated (right), 3-month-old mice. [file 12974_2019_1582_MOESM5_ESM.pdf]

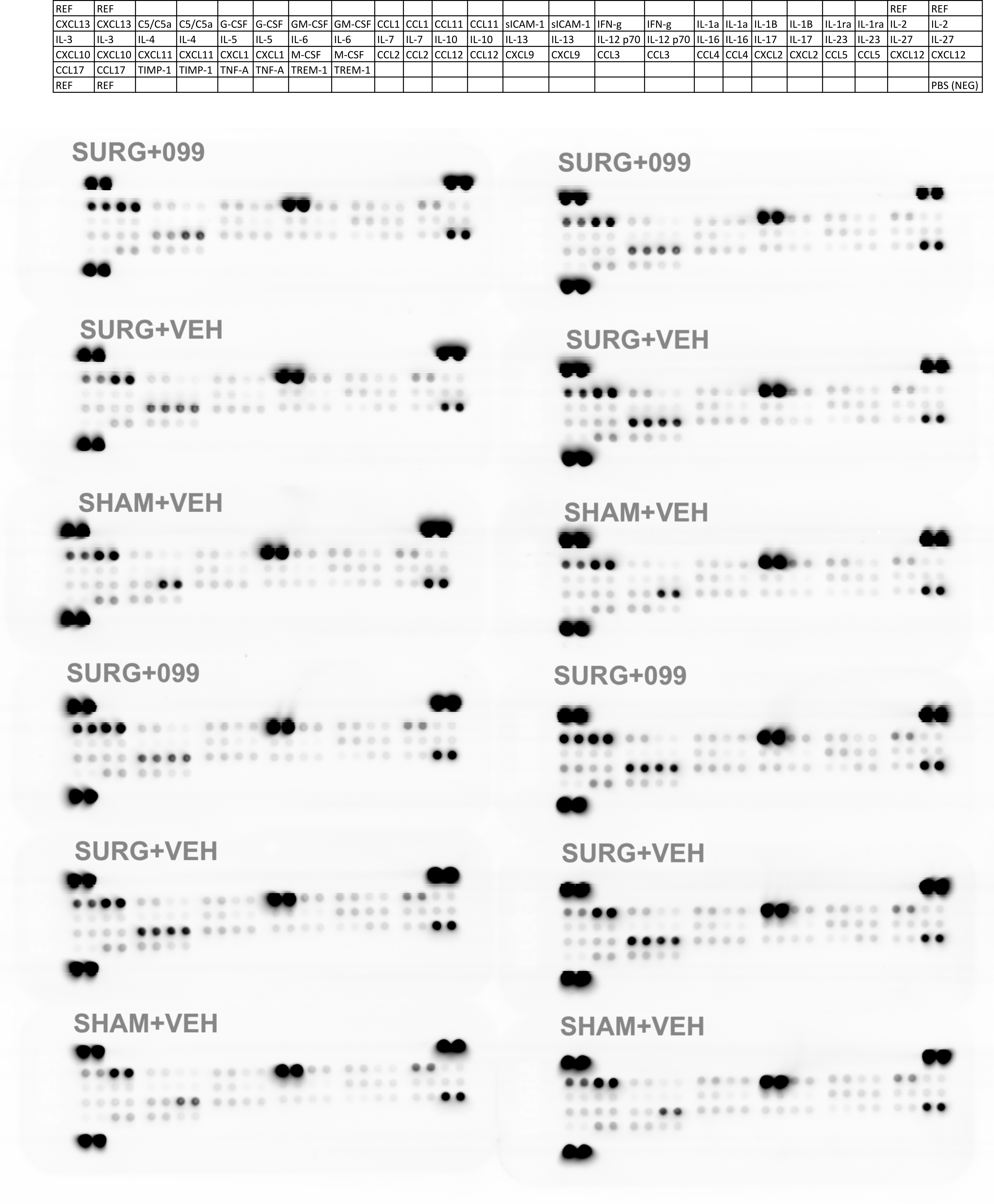

Supplement: Supplementary file 6 — Additional file 6: Figure S3. Images of Proteome Profiler cytokine arrays related to Figure 3. A key depicting the position of each analyte on each array is provided (top). [file 12974_2019_1582_MOESM6_ESM.png]

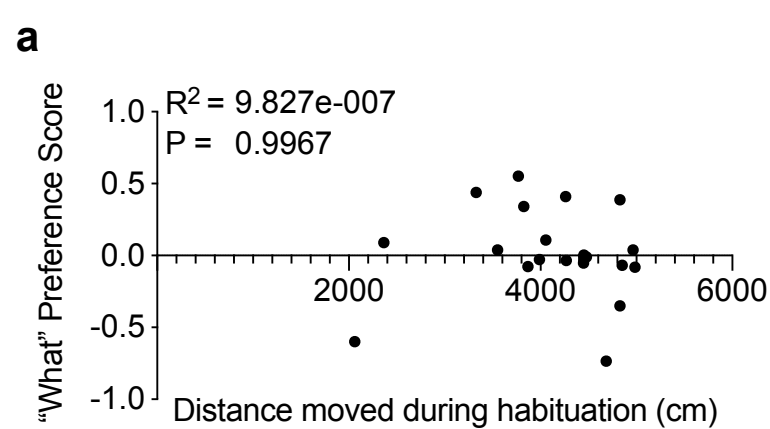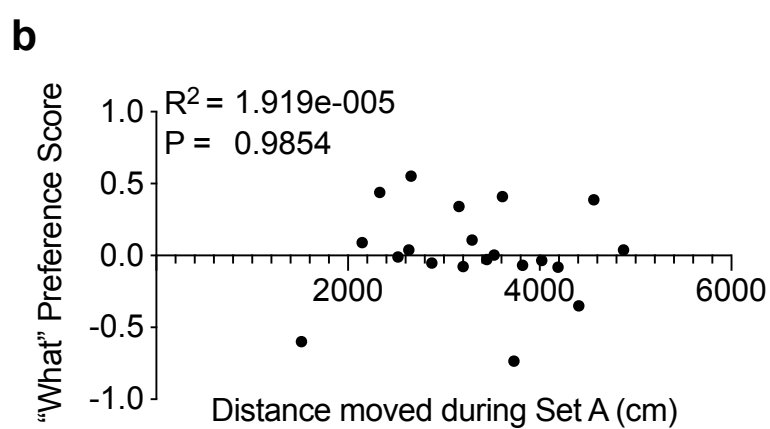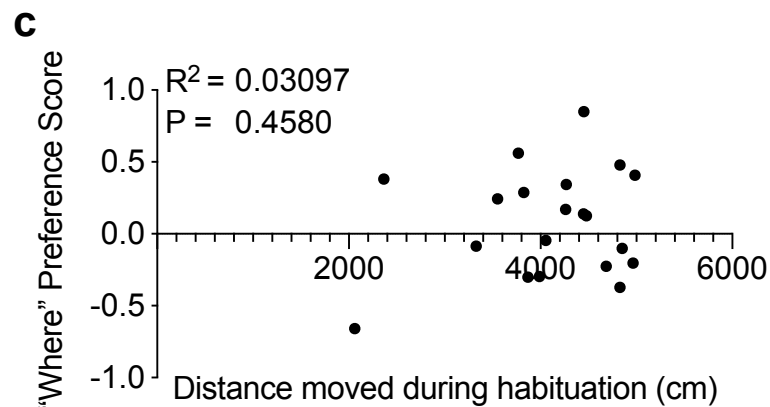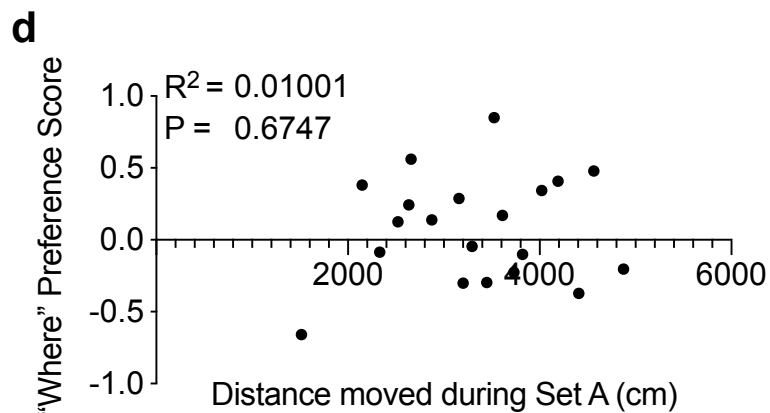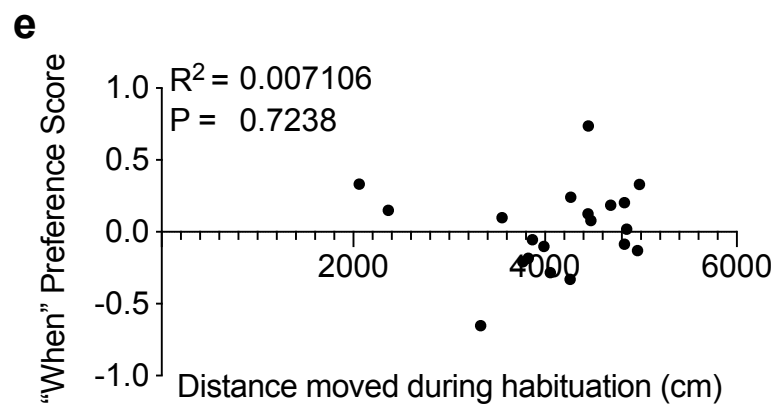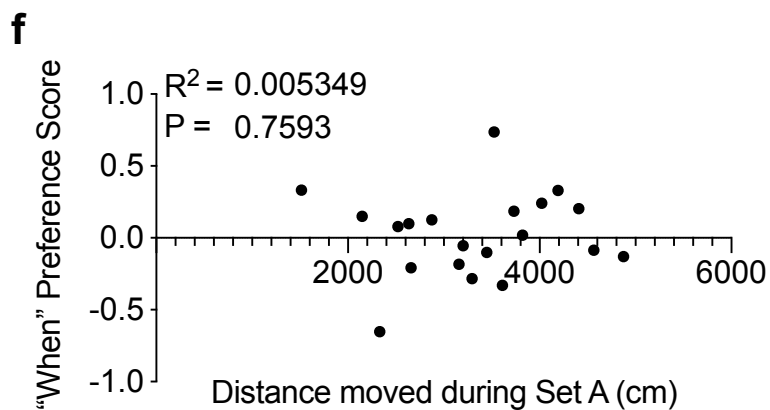

Supplement: Supplementary file 7 — Additional file 7: Figure S4. In surgical mice, distance moved during training is not correlated with behavioral performance in the “What-Where-When” object discrimination task (related to Figure 4). Pearson correlations; number of XY pairs per comparison = 20 (10 URMC-099 + Surgery, 10 Vehicle + Surgery). R2- and P-values (two-tailed) are shown for each correlation. [file 12974_2019_1582_MOESM7_ESM.pdf]

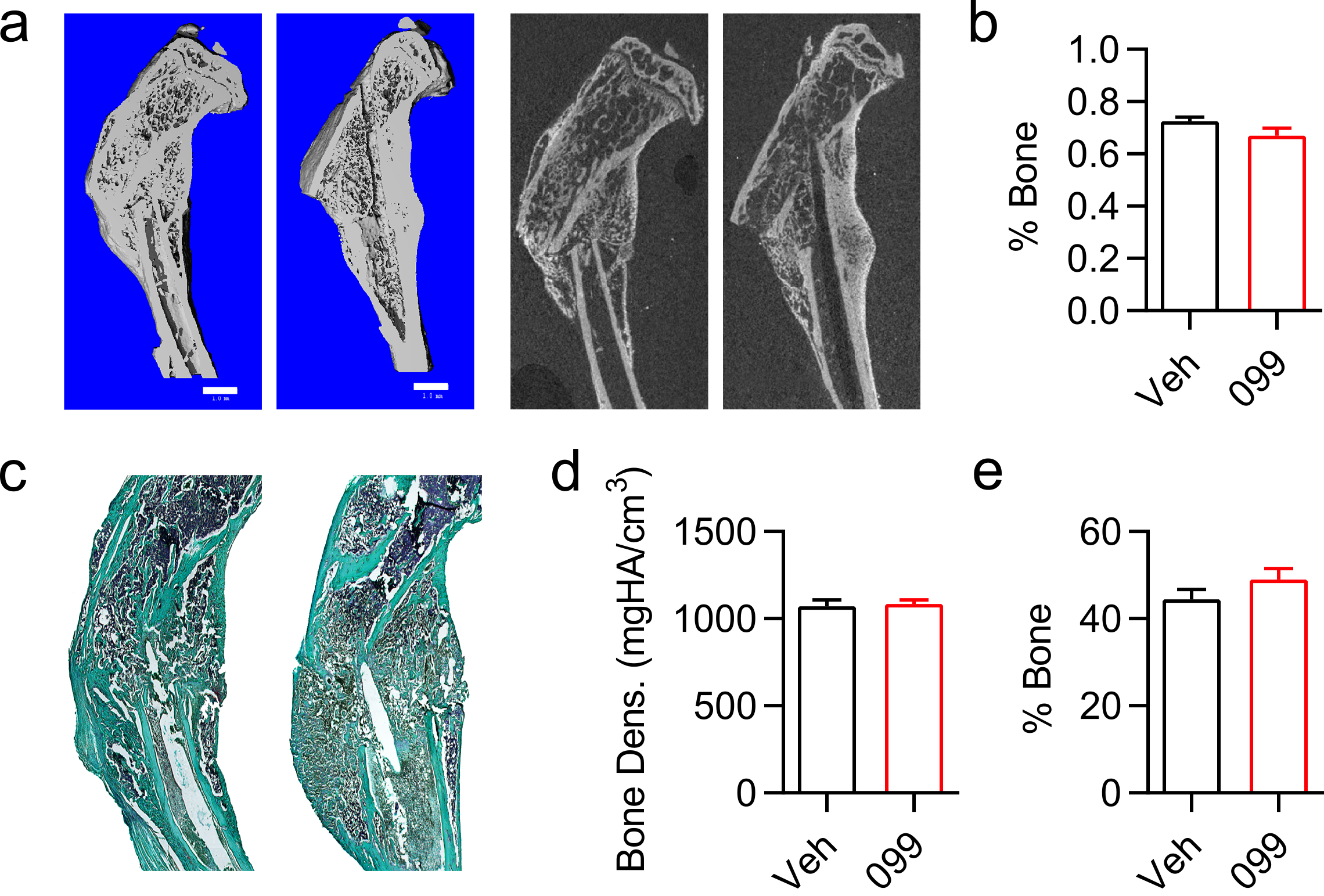

Supplement: Supplementary file 8 — Additional file 8: Figure S5. URMC-099 pre-treatment does not alter bone fracture healing. 3-month-old male mice were treated with either with URMC-099 or vehicle and fractures were induced surgically. Fracture calluses were investigated 21 days post-fracture to assess bone healing. (a-c) MicroCT was used to assess bone volume per total callus volume and bone mineral density. (d-e) Histomorphometry was used to determine the percent bone tissue within the fracture callus. N=6-7; results presented as mean ± SEM. Data analyzed by unpaired two-tailed t-test. [file 12974_2019_1582_MOESM8_ESM.png]
